# Surgical Management of Intrahepatic Cholangiocarcinoma: Quo Vadis

**DOI:** 10.3390/cancers15194691

**Published:** 2023-09-23

**Authors:** Dimitrios Moris

**Affiliations:** Department of Surgery, Duke University Medical Center, Durham, NC 27710, USA; dimitrios.moris@duke.edu; Tel.: +1-2165716614

Intrahepatic cholangiocarcinoma (ICC) is the second most common primary liver malignancy related to very high mortality rates. Management remains challenging due to highly heterogeneous disease biology, with most patients presenting with advanced disease and only 20–30% of patients being eligible for surgical resection, the only potentially curative treatment for most patients with locally unresectable or distant metastatic disease; survival remains limited to less than 1 year [1].

The success of surgical resection in ICC is dependent on various factors such as tumor differentiation, tumor size, tumor number, surgical margin, as extent of lymph node metastasis [2,3]. Tumor size is a critical determinant of prognosis after resection [4], even if there is no well-established cut-off that could be reliably used to guide decision-making and predict oncologic outcomes. Tumor Burden Score (TBS) as a single metric has recently been evaluated as a simple prognostic score of outcomes in many patients with liver malignancies, including patients with hepatocellular carcinoma (HCC) within and beyond Milan Criteria that could benefit from liver transplantation [5] or patients with “traditionally” unresectable HCC (Barcelona Clinic Liver Cancer (BCLC)-B stage) who can benefit from surgical resection [6,7]. In ICC, recent multi-institutional data report a potential role in predicting oncologic outcomes (5-year overall and recurrence-free survival) in patients undergoing curative resection of ICC. More specifically, higher TBS was related to significantly worse 5-year OS (low TBS: 48.3% versus high TBS: 17.3%, *p* < 0.001). Similarly, patients with low TBS had improved 5-year RFS compared with medium and high TBS patients (38.3% vs. 18.7% vs. 6.9%, *p* < 0.001) [8].

Since tumor size and surgical margins are strongly correlated with oncologic outcomes in patients with ICC undergoing resection, the extent of hepatectomy is a subject of ongoing discussion. Major anatomic hepatectomy is more frequently offered to ICC patients with large, multiple, and bilobar tumors with higher postoperative morbidity. Major hepatectomy had an equivalent overall and recurrence-free survival compared to minor hepatectomy [9]. These findings are similar to recent data reported from HCC literature [10]. Additionally, in patients with ICC and compromised liver function and overall performance status, elective major resection is related to a 10% 30-day mortality [11]. These findings further support the consideration of non-anatomic liver resections in patients with ICC in the frame of oncological equipoise.

The early recurrence is the major challenge that ICC patients undergoing resection face. Approximately 60% of these patients will recur within 2 years, while 25% of patients will recur in the first 6 months after curative-intent resection [12]. To optimize patient selection for resection, emerging multi-variable models focusing on readily available preoperative laboratory values have been proposed to identify individuals who will most benefit from surgical resection. Scores such as the Albumin-bilirubin ratio (ALBI) were recently shown to be a reliable marker of outcomes since patients with ALBI grade 2/3 were more likely to have eventful postoperative recovery, including higher 90-day mortality after ICC resection. Still, most importantly, median overall survival was inferior in patients with increased ALBI grade (grade 1, 49.6 months vs. grade 3, 16.9 months, *p* < 0.001). Similarly, the LabScore formula (8.2 + 1.45 × natural logarithm of carbohydrate antigen 19-9 + 0.84 × neutrophil-to-lymphocyte ratio + 0.03 × platelets—2.83 × albumin) was highly predictive of oncologic outcomes (0 to 9 with 54.9% 5-year overall survival, 10 to 19 with 38.2% and ≥20 with 21.6%, *p* < 0.001), with a c-index 0.70 that outperformed other scores such as prognostic nutritional index (c-index 0.58), and AJCC staging system (c-index 0.60) [13], Finally, scores that evaluate the baseline immune background and its role on oncologic outcomes such as the systemic immune inflammation index (SII) have been also used. In the ICC setting, patients with high SII had worse 5-year OS (37.7% vs. 46.6%, *p* < 0.001) and cancer-specific survival (46.1% vs. 50.1%, *p* < 0.001) compared with patients with low SII [14].

There is a universal understanding that the performance of predictive survival models for ICC is not ideal since comparisons of various predictive models showed that only the Wang model was the sole model with good performance (C-index above 0.70) for OS. This model incorporated common variables related to the success of ICC resection, such as tumor size and number, nodal metastasis, direct invasion into surrounding tissue, CA 19-9, and carcinoembryonic antigen (CEA) [15]. Hopefully, Artificial Intelligence and machine learning might offer more powerful and clinically meaningful models to predict outcomes in resectable patients with ICC. Models such as the Classification and Regression Tree (CART) have been recently used to capture homogeneous groups of patients undergoing surgery for ICC, showing that common variables such as tumor number and size, ALBI grade, and preoperative nodal (LN) status were the strongest of overall survival by describing four distinct groups of patients with statistically significant and clinically important different outcomes that included: (a) single ICC, size ≤ 5 cm, ALBI grade I, negative preoperative LN status; (b) single tumor > 5 cm or single tumor ≤ 5 cm with ALBI grade 2/3 or single tumor ≤ 5 cm with ALBI grade 1 and metastatic/suspicious LNs, (c) 2–3 tumors and (d) ≥ 4 tumors. The 5-year OS among groups was 60.5%, 35.8%, 27.5%, and 3.8%, respectively (*p* < 0.001). Similarly, 5-year RFS was 47%, 27.2%, 6.8%, and 0%, respectively (*p* < 0.001) [16]. Another machine learning analysis showed that ICC patients with small ICCs (median 4.6 cm) and median CA 19-9 and neutrophil-to-lymphocyte ratio (NLR) levels of 40.3 UI/mL and 2.6, respectively, had superior outcomes in terms of median overall survival compared to other groups with larger tumors or higher CA 19-9 or NLR values (60.4 months vs. 13.3 months, *p* < 0.001) [17].

Surgical Management of ICC is challenging due to the high incidence of recurrence and controversies around the management of nodal disease. Most current staging and predicting models are inadequate in predicting outcomes of patients with resectable disease. Most of them fail to identify patients with early recurrence potential who might benefit from alternative management strategies, including neoadjuvant chemotherapy.
